# Electromagnetic fields disrupt the pollination service by honeybees

**DOI:** 10.1126/sciadv.adh1455

**Published:** 2023-05-12

**Authors:** Marco A. Molina-Montenegro, Ian S. Acuña-Rodríguez, Gabriel I. Ballesteros, Mariela Baldelomar, Cristian Torres-Díaz, Bernardo R. Broitman, Diego P. Vázquez

**Affiliations:** ^1^ Centro de Ecología Integrativa (CEI), Instituto de Ciencias Biológicas, Universidad de Talca, Campus Talca, Talca, Chile.; ^2^ Instituto de Investigación Interdisciplinaria (I^3^), Universidad de Talca, Campus Talca, Talca, Chile.; ^3^ Grupo de Biodiversidad y Cambio Global (BCG), Departamento de Ciencias Básicas, Universidad del Bío-Bío, Chillán, Chile.; ^4^ Departamento de Ciencias, Facultad de Artes Liberales, Universidad Adolfo Ibáñez, Viña del Mar, Chile.; ^5^ Instituto Argentino de Investigaciones de las Zonas Áridas, CONICET, Mendoza, Argentina.; ^6^ Facultad de Ciencias Exactas y Naturales, Universidad Nacional de Cuyo, Mendoza, Argentina.

## Abstract

We assessed the effect that electromagnetic field (EMF) exerts on honeybees’ pollination efficiency using field and laboratory experiments. First, we measured levels of gene and protein expression in metabolic pathways involved in stress and behavioral responses elicited by EMF. Second, we assessed the effect of EMF on honeybee behavior and seed production by the honeybee-pollinated California poppy and, lastly, by measuring the consequences of pollination failure on plants’ community richness and abundance. EMF exposure exerted strong physiological stress on honeybees as shown by the enhanced expression of heat-shock proteins and genes involved in antioxidant activity and affected the expression levels of behavior-related genes. Moreover, California poppy individuals growing near EMF received fewer honeybee visits and produced fewer seeds than plants growing far from EMF. Last, we found a hump-shaped relationship between EMF and plant species richness and plant abundance. Our study provides conclusive evidence of detrimental impacts of EMF on honeybee’s pollination behavior, leading to negative effects on plant community.

## Teaser

Electromagnetic fields exert a negative effect on honeybee pollination, which has consequences for the plant community.

## INTRODUCTION

Human activities have altered natural ecosystems worldwide in multiple ways through habitat destruction and degradation, biodiversity loss, and changes in species interactions (*1*). These changes to natural ecosystems have also impaired the goods and services provided by ecosystems to humanity, ultimately hindering human welfare (*2*–*5*). Pollination stands out among the ecosystem services threatened by human activities (*6*), especially through the transformation of natural vegetated areas into cities and croplands (*7*).

As landscapes underwent transformation, access to electric energy increased from ca. 70% of the world population at the late 1990s to ca. 90% today (*8*). Concomitantly wild organisms are increasingly exposed to the electromagnetic fields (EMFs) from distribution networks (*9*). The concern about the detrimental effects of EMF exposure on wild organisms is now reflected in the growing number of articles on the topic (*10*).

Studies assessing the effects of EMF on organisms range from vertebrates (*11*, *12*) to invertebrates (*13*, *14*) and humans (*15*, *16*). These studies indicate that EMFs lead to a higher mortality of different organisms, which may vary according to the identity of the taxa studied and the conditions of exposure to EMF, such as intensity, time, and source of exposure. In particular, in insects, EMF can directly hamper development, survival, and navigation in fruit flies (*17*–*19*) and honeybees (*20*), ultimately decreasing the abundance of these wild insect pollinators by negatively affecting their fitness [e.g., (*21*)]. By affecting pollinators, EMF might also disrupt plant-pollinator interactions and pollination services, although no previous studies have addressed this potential effect or assessed the mechanisms behind the negative effects exerted by EMF on pollinators.

The honeybee (*Apis mellifera* L.) is the most frequent floral visitor in natural habitats worldwide, because of its wide human-driven distribution, generalist foraging (outdoor food search), and pollination effectiveness (*22*). Hence, foraging bees must navigate successfully to locate food sources and return to their nests using the natural horizontal component of Earth’s EMF as cues (*20*). This requires a highly sensitive magnetoreception system based on light-independent, ferromagnetic-based proteins and radical pair–based chemical magnetoreceptors, such as the photosensitive cryptochrome 2 (Cry2), which is involved in sensing the directional component of EMF (*23*). Thus, honeybees are adapted to fluctuations of the natural EMF emitted by lightning, animals, and extraterrestrial sources, such as sunspot solar cycles and solar geostorms (*20*, *24*).

However, honeybees are also being increasingly exposed to artificial, low-frequency EMF (such as those from overhead power lines), which acts as a stressor on honeybees, by altering the magnetic maps used during foraging flights and navigation and producing a magnetoreception disorder (*20*). This leads to fewer honeybees returning to the colony, disorientation, or even a total loss of adult foragers (colony collapse disorder) (*25*). These negative effects of EMF could cascade into a number of additional effects on insects’ physiology and behavior, including less pollen and honey harvested (*26*, *27*), impaired learning ability, flight dynamics, foraging, and feeding (*28*), as well as increased piping in the colony (*29*). Hence, perturbations in terms of EMF from anthropic sources would disrupt the pollination services provided by honeybees, as they would avoid places exposed to EMF (*24*). However, no previous studies have addressed the consequences of honeybees’ exposure to EMF in terms of (i) plant pollination and reproduction and (ii) biochemical and molecular mechanisms underlying honeybee behavioral and antioxidant stress responses.

**Fig. 1.**
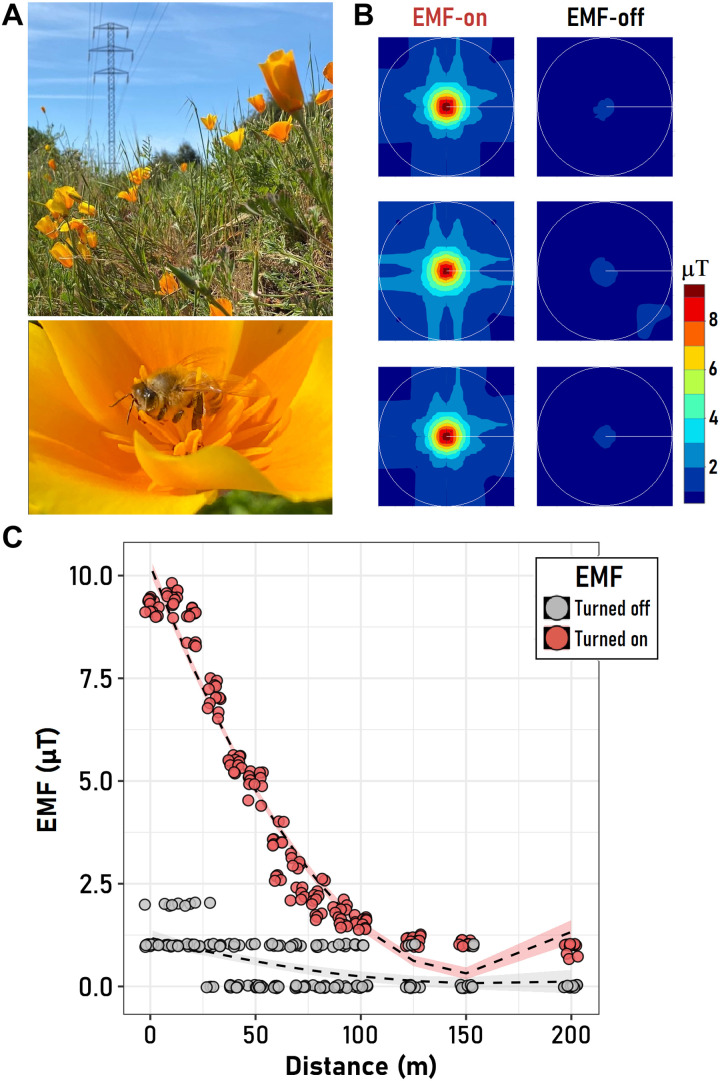
Characterization of EMF in the field and target species. (**A**) View of the field populations of *E. californica* and a honeybee visiting an individual flower during field surveys. (**B**) Interpolated field pattern of the EMF radiation gradient measured around high-voltage towers on eight orthogonal directions in either inactive (EMF-off) or active (EMF-on) transmission lines; the white circle inside each plot indicates the perimeter of a 200-m-radius circumference around the sampled tower. The relation between the distance to the electric infrastructure (towers) and the intensity of the EMF is presented in (**C**), together with the fitted LMM for the measurements collected at inactive (gray) or active (red) towers. The shaded area represents the 95% confidence interval for the respective fitted model.

In this study, we used a combination of observational field studies and experimental manipulations using a purpose-built solenoid to simulate and assess the impacts of EMF induced by six high-voltage towers on honeybee’s physiology, behavior, and pollination service on the self-incompatible herbaceous plant *Eschscholzia californica* (California poppy) (Fig. 1A). The towers selected for this study were tall structures (20 m of height), built mainly with steel and some parts of copper, used to support a high-voltage overhead power line with an energy box storer in the upper section of the tower. These devices generate an EMF close to 10 μT, with a peak recorded between 12 and 17 m from the base of the tower and at 25 to 30 cm of height, and decrease to almost extinction at 200 m from the base of the tower (Fig. 1, B and C). In addition, we assessed the expression levels of selected candidate genes involved in antioxidant defense, foraging, spatial learning, and magnetoreception, as a possible mechanism behind the negative effects of EMF on honeybees. Specifically, we hypothesized that (i) EMF modulates the expression levels of genes linked with oxidative stress pathways, foraging behavior, learning, and magnetic navigation; (ii) EMF exerts physiological stress in honeybees, disrupting their behavior, thus their pollination service, reducing the female reproductive success in a target plant species in the field; and (iii) the effects of EMF on plant reproduction will cascade through the community, affecting overall plant abundance and species richness. We predicted that honeybees exposed to EMF should show increased biochemical stress markers and changes in gene expression levels linked to both stress and foraging, thus affecting their pollination service compared to honeybees unexposed to EMF. In addition, plants growing under the influence of EMF in the field will receive fewer honeybee visits, which should, in turn, reduce their reproductive output. Last, plant species abundance and richness around towers that are actively transmitting electric energy should increase with the distance from the tower infrastructure, a gradient that will be absent around inactive (i.e., not transmitting) towers.

## RESULTS

As expected, measurements in the field showed that the intensity of the EMF was consistently low (~1.5 μT) around inactive infrastructure (“EMF-off”), while around active transmission towers, the intensity was 10× higher and decreased with distance from the source (“EMF-on”; Fig. 1B). At 10 m from active towers, the mean intensity of the EMF was 9.47 μT (± 0.21 SD), dropping to half of its intensity at 50 m and becoming virtually undetectable at 200 m (Fig. 1C). The strength of the EMF did not vary significantly with cardinal direction or between sites (table S1), suggesting that transmission towers produced consistent EMF intensities with an orthogonal spatial distribution around them (Fig. 1, B and C).

**Fig. 2.**
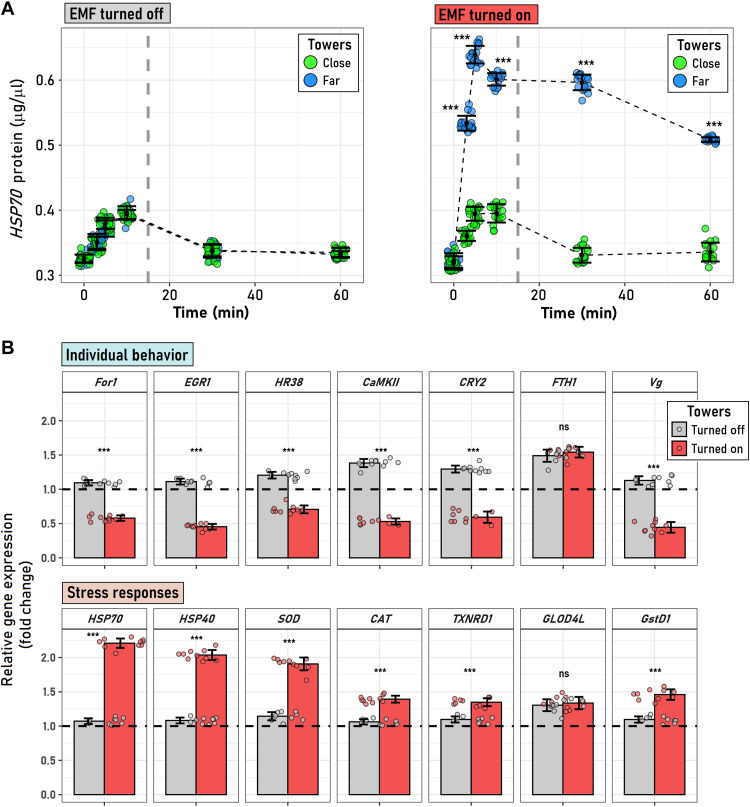
Effects of EMF on honeybee physiology and gene expression levels. (**A**) Temporal variation of the individual concentration of the Hsp70 protein in the body tissue of honeybees exposed (or not) to an EMF around high-voltage transmission tower. Temporal Hsp70 variation was monitored for 60 min on *A. mellifera* individuals that were maintained for 15 min (vertical gray dashed line) close to (15 to 25 m) or far from (210 to 235 m) towers, which were either transmitting current (EMF turned on) or not (EMF turned off). (**B**) For honeybees exposed to EMF generated by solenoids under laboratory conditions, we also examined the relative expression of 14 genes. Relative gene expression levels under EMF-off versus EMF-on conditions. The first row shows results for seven genes related with the behavior of *A. mellifera* [foraging (*For1*), early growth response gene type 1 (*EGR1*), hormone receptor 38 (*HR38*), calcium/calmodulin-dependent protein kinase II (*CaMKII*), *CRY2*, ferritin heavy polipeptide 1 (*FTH1*), and vitellogenin (*Vg*)], while the second row shows seven genes linked with physiological stress [*HSP70*, *HSP40*, superoxide dismutase (*SOD*), catalase (*CAT*), thioredoxin reductase 1 (*TXNRD1*), glyoxalase domain–containing protein 4–like (*GLOD4*), and glutathione *S*-transferase D1 (*GstD1*)]. Error bars in both (A) and (B) represent ±SD. *** in (A) highlight significant differences (*P* < 0.0001) between individuals maintained close to and far from the towers at a given time, as estimated by an a posteriori Tukey honest significant difference test at *P* = 0.05. In (B), asterisks point to significant differences between the group averages (EMF-off versus EMF-on) according to a two-sample *t* test (*P* < 0.0001). The horizontal dashed line in (B) denoted the onefold expression level; independent one-sample *t* tests realized on each gene expression denoted that all genes were either significantly overexpressed (bar above 1) or repressed (bar below 1). ns, not significant.

When we investigated the effect of EMF under field conditions, we found that the synthesis of the heat-shock protein 70 (Hsp70) stress biomarker protein was significantly higher among honeybees maintained close to the transmission towers (10 to 25 m) compared with those far from them (210 to 235 m) but only when the high-voltage infrastructure was actively transmitting energy (Fig. 2A and table S2). Expression levels of Hsp70 doubled after 5 min in honeybees close to the active transmission towers when compared to the level of expression observed in honeybees positioned away from them. Honeybees positioned close to the inactive towers and far from them showed the same response (Fig. 2A). Differences in the level of synthesis of the Hsp70 between honeybees close to and far from towers that were actively transmitting energy tended to decrease when both groups were retrieved from the field (after 15 min), yet significant differences between treatments remained 45 min after the exposure (Fig. 2A).

Subsequently, we aimed at falsifying the conditions observed in the field and seek evidence of a stress response at the genetic level linked to the exposure of honeybees to EMF. To this end, we designed an experimental treatment using a purpose-built solenoid to expose individual honeybees to the EMF conditions observed in the field. For 12 of the 14 evaluated genes, a significant differential expression was observed between unexposed (EMF-off) and exposed honeybees (EMF-on; Fig. 2B). The gene expression effect was consistent among the two functional gene groups included in our assessment (behavioral and stress-response genes). Each functional group respond in similar ways depending on the experimental conditions, while for honeybees maintained in the inactive solenoid (EMF-off), all expression levels were slightly overexpressed (relative to a normalizer gene); for those in the active device (EMF-on), most behavior-related genes appeared significantly repressed (six of seven genes), and most stress-response genes were consistently overexpressed (six of seven genes; Fig. 2B and tables S3 and S4).

Among the honeybees of the solenoid experiment exposed to an active EMF, the average concentration level of Hsp70 in their bodies (~0.68 ug/μl) was a 52% higher relative to nonexposed honeybees (fig. S1 and table S5), and similar to the peak shown in the field, honeybees maintained close to towers that were actively transmitting current (~0.63 ug/μl). In addition, honeybees maintained in the inactive solenoid showed similar Hsp70 concentrations at the start and end of experimental treatment (3 min). Thus, experimental EMF treatment allowed us to falsify the effects of an EMF in the field and the procedural control; manipulating and maintaining honeybees inside the inactive experimental EMF treatment did not generate an evident stress (fig. S1).

**Fig. 3.**
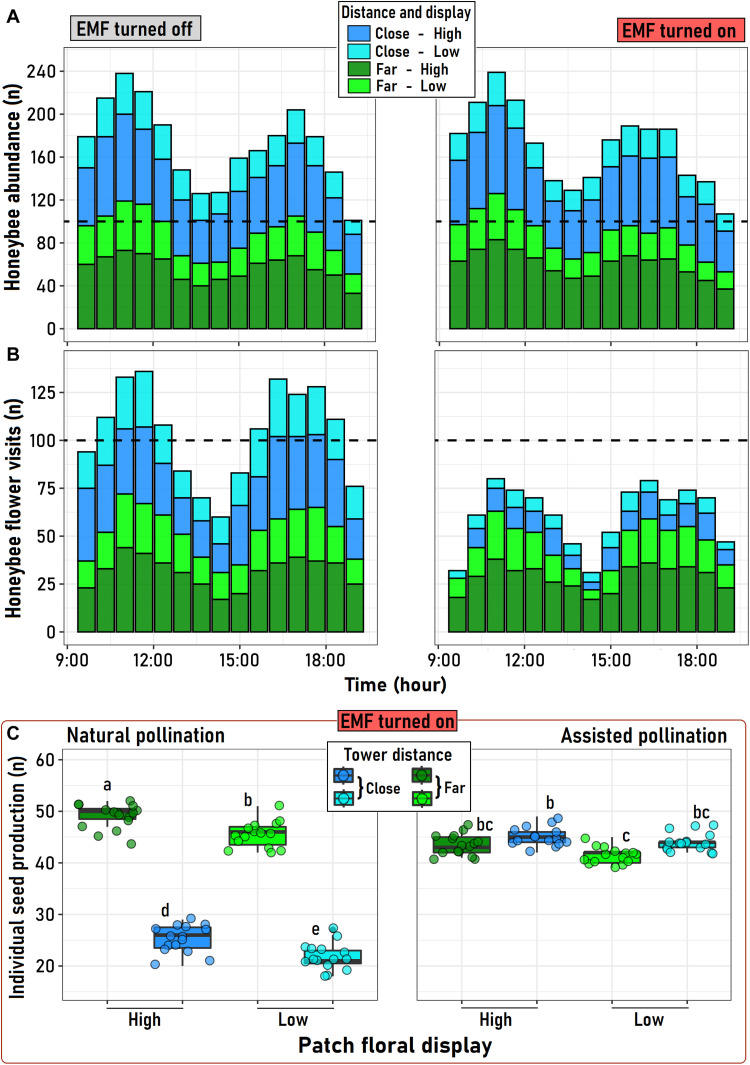
Effects of EMF on pollination services and plant reproduction. Distribution of the abundance of *A. mellifera* individuals (**A**) and their flower visit count on California poppy patches during daytime (**B**) as a function of the patch floral display (low, yellow green/cyan; high, green/blue) and the distance (blue/cyan, close = 15 to 25 m; yellow green/green, far = 210 to 235 m) at which floral patches were from the electrical current transmitting towers. The bars represent the summation of all observations recorded for three consecutive days at towers that were not transmitting any current (EMF turned off) and at towers that did (EMF turned on; see Materials and Methods for details). The dashed line at 100 counts is for reference at the bottom (**C**), the seed production of *E. californica* individuals with different type of pollination (natural or assisted) among the four experimental plant groups (colors) under active transmission lines (EMF-on). Points represent patch values at a given hour in (A) and (B) (3-day summation) and individual values at (C); boxes and bars behind the points at (C) correspond to the group interquartile distribution (5, 25, 50, 75, and 90 intervals). Letters above each group represent the results of the marginal mean paired comparison performed on the data. Similar letters indicate nonsignificant differences (*P >* 0.05) between groups.

In the field, total honeybee abundance and honeybee visitation frequency to flowers of California poppy were higher under high flower density than under low floral density, regardless of the distance to transmission towers (Fig. 3). Honeybee abundance did not change significantly with distance to transmission towers, both for active and inactive (switched off) towers (Fig. 3A). However, honeybee’s visitation frequency to California poppy flowers growing far (210 to 235 m) from the base of active towers was ~16% lower than for inactive towers (Fig. 3B). Likewise, honeybee visitation to California poppy growing close (10 to 25 m) to the active towers decreased sharply (~308%) compared to inactive towers (Fig. 3B).

Statistical fitting [assessed by general additive mixed model (GAMM)] of the temporal dynamics of honeybee abundances around the transmission towers revealed that spatial patterns were mainly determined by the floral display of the California poppy individuals and not by the distance to the transmission towers, a pattern that was indistinctly observed despite the activity of the transmission towers (EMF-off and EMF-on; table S6). Thus, in both scenarios of EMF activity, honeybee abundances were greater around individual plants with high floral displays independently of their distance (close or far) to the towers (fig. S2A). In turn, besides floral display, the distance to the towers also affected the rates and dynamics of flower visitation by honeybees, an effect that we only observed in Californian poppy patch located around active transmission lines (fig. S2B and table S7). Accordingly, the expected disruption of EMF on honeybee activity around active transmission towers mainly occurred close to these structures (significant “Distance × Display” interaction; table S7): Individuals with high floral display growing close to the towers (close and high) received, in average, fewer visits during the day than plants with low floral display but growing far from them (far and low). The limited honeybee visits experienced by *E. californica* close to the active towers was also observed in the temporal homogenization of the rates of honeybee visits received by those individual plants along the day (nonsignificant smooth GAMM terms; table S7). In this sense, the expected peaks of daily activity, common among insects at the morning and afternoon, were only observed for honeybees surveyed around inactive towers, or else, those located far from the towers with active energy transmission (fig. S2B).

Having established the strong physiological and behavioral effects of EMF on honeybees both in the laboratory and in the field, we examined the downstream ecological effects by manipulating and quantifying seed production in the field. For plants located around towers actively transmitting energy, the distance to the towers (close or far) had the strongest effects on seed production, followed by pollination type and floral display size (Fig. 3C and table S8). Seed production varied more in plants that were naturally pollinated than in plants that were manually pollinated (Fig. 3C). The negative impact of being close to the towers on plant reproduction was evident only in the natural pollination treatment (Fig. 3C), which is consistent with the significant interaction between those factors (table S8). In contrast, the interaction between floral display and pollination type resulted to be nonsignificant (table S4) probably because of the high variability introduced by the distance to the EMF source on natural seed production (Fig. 3C). In contrast, when pollination was manually assisted, no differences in seed production between plants at different distances from the towers or with contrasting floral display levels were found, indicating that assisting pollination erased the impact of EMF on seed production (Fig. 3C).

**Fig. 4.**
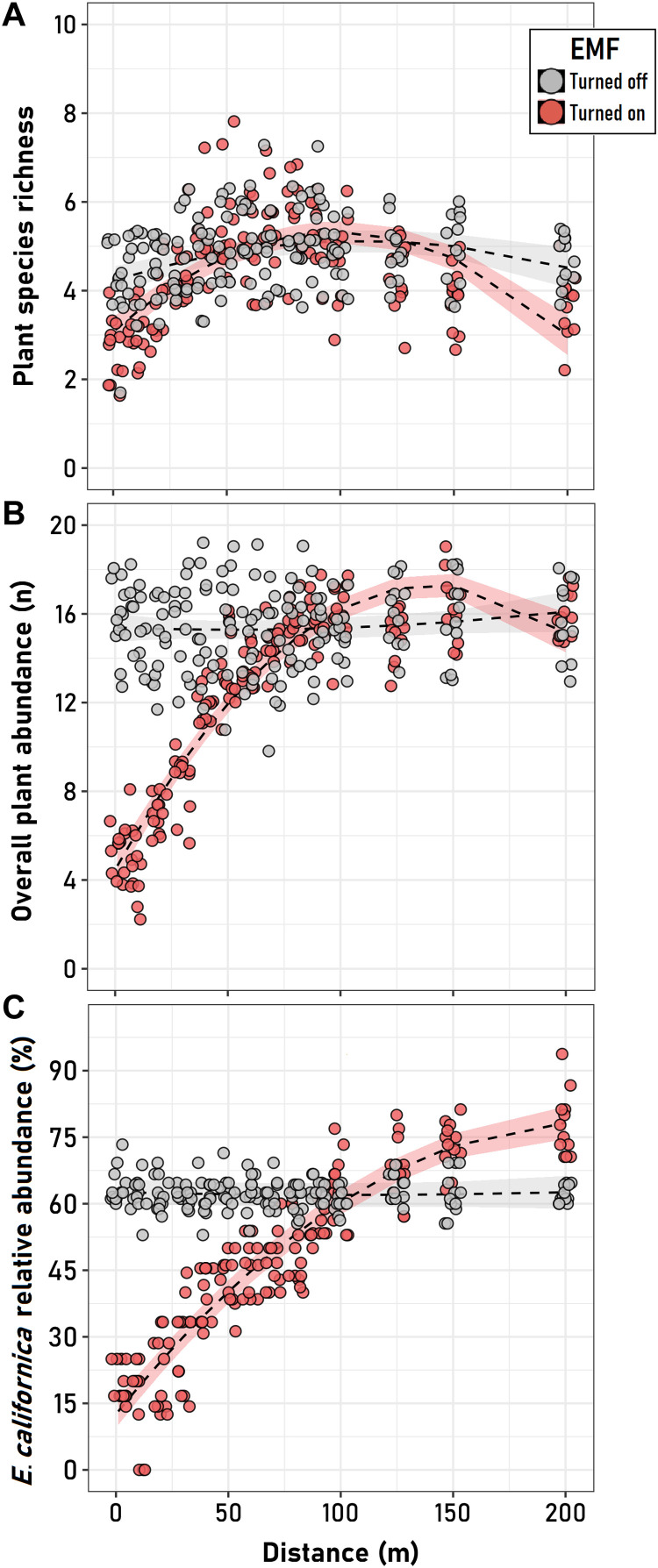
Effects of EMF on plant community. Relationships between the distance to the transmission towers and (**A**) plant species richness, (**B**) overall plant species, and (**C**) *E. californica* relative abundance assessed at towers that were not transmitting any current (EMF turned off, gray) and at towers that did (EMF turned on, red). Trend lines were fitted according to quadratic models considering the site and the cardinal orientation around the tower as random factors. The shaded area represents the 95% confidence interval for the respective fitted model. For all response variables, the EMF factor (turned off/turned on) resulted to be significant in explaining the variance of all fitted models.

Concerning the *E. californica* population and its associated plant community, we observed that EMF significantly affected the three spatial patterns estimated in our study: species richness distribution, overall abundance, and the relative abundance of *E. californica,* which were tightly coupled on the activity of transmission towers (Fig. 4 and table S9). The fitted linear mixed models (LMMs) suggest that all three plant community variables were significantly affected by the activity of the transmission towers (EMF-off/EMF-on), a factor that was significant in all models (Fig. 4). However, while the quadratic model accurately described the spatial organization of all three variables around active towers (EMF-on), it was significant only for species richness around inactive towers (EMF-off). In this last case, besides a small but significant trend of increased species richness away from the towers (the linear predictor), plant communities around both inactive (EMF-off) and active (EMF-on) towers showed a humped-shaped response (the quadratic term), which was more pronounced around active towers (table S9). By contrast, for overall community abundance and for the relative abundance of the California poppy, the humped-shaped (quadratic) term of the spatial distribution around the electrical infrastructure was only present under the EMF-on condition (Fig. 4). The linear (i.e., constant) distribution of these variables around inactive towers forced the nonsignificance of the distance as a main factor in their respective models. However, as their significant interactions with EMF activity show, the effect of the distance on the overall community abundance and relative abundance of the California poppy is only evident around active (EMF-on) towers (Fig. 4).

## DISCUSSION

We have shown that the presence of EMF under field conditions significantly impaired honeybees’ pollination services to plants following a putative molecular mechanism associated with behavioral and physiological stress. Individual stress processes were driven by the activity of specific genes, an effect that we quantitatively reproduced under laboratory conditions. The organismal-level impacts translated into a lower number of floral visits that reduced seed production, which, in turn, lowered diversity and plant abundance. The negative effects, from genes to experimental plant communities, were chiefly related to distance to the source: They were observed only when transmission towers were online (thus emitting EMF).

We propose that honeybees’ exposure to EMF disturbs their foraging capabilities by altering their magnetic navigation, learning, decision-making mechanisms, flight, and foraging, thus impairing pollination activity (*30*, *28*). This hypothesis would explain the observed reduction of workers’ flower visitation around areas located in the proximity of active electric transmission towers, which we have established to be a prominent source of stress for honeybees (*28*). After exposure to EMF, we found a significant induction in terms of expression profiles of genes encoding for different antioxidant-related proteins underpinning physiological stress when compared with the nonexposed conditions, where honeybees’ physiological stress was not apparent. The substantial increase in the activity of the biochemical stress pathways would allow individuals to counteract the potential production of radical pair intermediates with a highly reactive oxygen nature upon magnetic field–induced stress (*31*). Thus, proteins, such as catalase, thioredoxin reductase 1, superoxide dismutase, Hsp40, and Hsp70, among others, would effectively prevent the damaging effects of oxidative stress derived from harmful electronic interactions (*31*). These genes have been widely used as indicators of multiple stressors (*32*), including exposure to EMF on other insect species (*31*). Moreover, higher levels of Hsp70 production in honeybees after exposition to EMF were evidenced, under both field conditions and manipulative experiment in laboratory, suggesting that honeybees are experiencing the effects of oxidative stress induced by an exposure to an anthropic EMF (*33*).

We also observed that EMF significantly down-regulated the expression levels of selected candidate genes underpinning the age-related transition from working in the hive (nurse) to foraging for food outside (*34*). These include early response gene 1 (*ERG1*), hormone receptor 38 (*HR38*), guanosine 3′,5′-monophosphate–dependent protein kinase (foraging, *For1*), vitellogenin (*Vg*), calcium/calmodulin-dependent protein kinase II (*CaMKII*), and *Cry2*. Therefore, EMF may have a negative impact on long-term memory formation (*35*), impairment of magnetic orientation in white light (*36*), task specialization (*37*), reduced locomotor activity, and reversion to a nurse phenotype (*34*).

Together, these results provide a strong indication that exposure to EMF during extranidal food gathering induce a substantial stress on honeybee physiology, presumably due to an increase in cell temperature and brain tissue damage during exposure to EMF (*38*). In insects, even a small increase in body temperature induces alterations in the respiration metabolism and can affect the functioning of the nervous and endocrine systems, as reflected in the behavioral responses of honeybees and underlying changes in terms of gene and protein expression levels (*39*). Nonetheless, further research using a transcriptomics/proteomics and biochemical approach would be required to unveil the extent of the impacts of EMF on insect pollinators. This knowledge would help to get a wider perspective on the still unforeseen consequences of human activity on animals and plants.

As observed around both the inactive and active towers, honeybee abundance was more influenced by floral display than by the distance to the towers, being greater at higher densities of California poppy and independent from the tower proximity. Similar results were found for wild above-ground nesting bees (*21*), where pollinator species richness was unaffected by EMF. However, previous studies did not consider plant display, which, in our study, showed an effect in the pollinator abundance, irrespective of the distance to the active towers. A high floral display may determine great visitation rates by potential pollinators; our results indicated that the negative effect of EMF is so strong that even patches with low floral abundance but away from the infrastructure can receive more pollinator visits than patches with greater floral display but close to the EMF source. Thus, although EMF did not affect honeybee abundance in the study area, it was associated with decreased number of honeybees contacting the flowers of California poppy individuals, independently of floral abundance. A plausible explanation for this result has to do with decreased cognitive and motor abilities (*28*) and orientation capacities (*25*) reported for honeybees as a consequence of exposure to EMF. Decreased pollinator visitation matched a significant decrease in plant reproductive success. We found no significant differences in seed production among treatments for flowers that received hand pollination, independently if were exposed or not to effects of the EMF. Hence, our results indicate that an impaired pollination service is the most parsimonious explanation for the observed reductions in seed production than any potential direct effect of the EMF on the plants through processes such as ovule abortion or reduced pollen germination. This finding contrasts with previous studies documenting negative direct impacts of EMF on plant reproduction through decreased pollen germination (*40*). Thus, our results suggest that the main impacts of EMF on plant reproduction are mediated by indirect effects on pollinator behavior and, therefore, shall be greater for self-incompatible plant species (*41*).

The negative impacts of EMF on honeybees described here could produce substantial reductions in plant reproduction at local scale, i.e., around transmission lines. Considering that the global increase in agricultural production (>300%) required for global food security depends on animal pollination (*42*), some studies (*43*) have estimated that without pollination services by bees, the total agricultural production would decrease by 8%, with a large fraction of the impact taking place in developing countries. Honeybees are the main pollinator species of our study species and of many staple crops (*44*). Currently, the numbers of honeybee colonies are declining in some parts of the world because of a wide variety of threats, such as agrochemical poisoning, invasive species, climate change, and habitat fragmentation (*6*). In this context, EMF represents an emerging threat that is increasing in its footprint through agricultural landscapes (*9*). The interaction between all these threats could further threaten honeybee populations, thus leading to production losses for several crops (*44*), making the challenge of achieving global food security more difficult.

In summary, our findings support the notion that EMF can have direct negative impacts on pollination service by honeybees, with detrimental consequences on the seed output of insect-pollinated plant species and indirect negative impacts on plant community (abundance and richness) due to possible impairment in the pollination service required by the plant community (*40*). We also highlight that the magnitude of the impact of EMF on pollination service, at local scale, can be much greater than previously thought. Honeybees use electric fields for intraspecific (within hive) and interspecific (plant-pollinator) communication (*9*) and are able to detect the anthropogenic EMF, and their capacity for orientation, navigation, and foraging is being impaired, which would ultimately affect their health and survival (*9*, *28*, *45*). Our study provides strong evidence of detrimental effects of EMF on honeybee’s visitation and plant reproduction and may contribute to explaining, at least in part, the global pollination crisis that risks the adequate production of many crops.

## MATERIALS AND METHODS

### Study site and species

We conducted the study in Quinamavida (35°48′S, 71°25′W), in the Maule region, Chile. The climate is Mediterranean, with an average of 686 mm of annual rainfall, concentrated mainly in the austral winter months (May to August) and warm, dry summers (*46*). At the study site, mean annual temperature is 14.7°C ranging from 19.8° and 7.3°C (data extracted from the Center for Climate and Resilience Research database; https://explorador.cr2.cl). Vegetation corresponds to a Mediterranean sclerophyllous forest dominated by trees, such as *Lithrea caustica*, *Maytenus boaria*, *Peumus boldus*, and *Quillaja saponaria*, and medium-size shrubs, such as *Baccharis* spp. and *Renatilla trinervia*. The herbaceous vegetation corresponds to an ephemeral community, consisting of grasses, geophytes, and several annual species with showy flowers, such as *Alstroemeria* spp. and *Eschscholtzia californica* (*47*).

*E. californica* is a perennial, self-incompatible plant pollinated mostly by bees with *Apis* and *Bombus* bee species representing the most frequent pollinators (*48*). Honeybees represented the most frequent visitors with 88% of all visitors of this species in our study site, followed by other three visitors with a presence of less than 7% for each one (Fig. 1A and table S10). The California poppy is native to the United States and invasive in Chile, New Zealand, Australia, and South Africa (*49*) and has colonized successfully a wide range of environmental conditions in both its native and introduced ranges, often occupying both natural and human disturbed open landscapes (*48*, *50*). In Chile, this species has a broad elevational and latitudinal range (*51*).

### EMF characterization

The study site harbors transmission lines and towers devoted to energy transmission or mobile phone infrastructure. Current regulations mandate the strip underneath high-voltage power lines to be kept clear from large trees around an area as wide as the height of the transmission lines. However, we found no evidence of clearing or fire management activity against herbaceous plants or trees, either underneath or around the towers and power lines at the study sites, that could affect the abundance and distribution values of plant species within this community.

To estimate the intensity of the EMF, we selected three transmission towers of 20 m of height located at least 500 m away from each other (hereafter, EMF-on). From the base of every tower and toward each of eight cardinal points conformed by the equivalent orthogonal axes at 0° (facing north), 45°, 90°, 135°, 180°, 225°, 270°, and 315°, we established a 200-m transect and recorded the EMF intensity (in microtesla) every 25 m at 50 cm from the ground using an EMF meter (model TM191, Tenmars Electronics Co. Ltd., Taiwan). The same exercise was performed on an additional group of three towers that were part of an inactive section of the transmission line (hereafter, EMF-off); hence, they acted as our experimental control to assess the effect of the own structure (transmission tower) in our analyses.

### Effects of EMF on honeybee physiology and gene expression levels

To assess the EMF effects on the physiology, stress, and behavior of honeybees under both field and laboratory conditions, we recorded the differential synthesis of heat-shock proteins (Hsp70), a recognized marker of physiological stress in bees (*52*), and two groups of genes. The two functional gene groups included in our assessment corresponded to those related with behavior genes: foraging (*For1*), early growth response gene type 1 (*EGR1*), hormone receptor 38 (*HR38*), calcium/calmodulin-dependent protein kinase II (*CaMKII*), *CRY2*, ferritin heavy polipeptide 1 (*FTH1*), and vitellogenin (*Vg*); and stress-response genes: *HSP70*, *HSP40*, superoxide dismutase (*SOD*), catalase (*CAT*), thioredoxin reductase 1 (*TXNRD1*), glyoxalase domain–containing protein 4-like (*GLOD4*), and glutathione-*S*-transferase D1 (*GstD1*). We sourced all honeybees used for experimentation from a honeybee-keeper located near the study site and raised individuals from the same hive and cohort to avoid genetically based confounding factors.

First, in the field, either inactive (EMF-off) or active (EMF-on) transmission lines, we placed honeybees in 1000-cm^3^ (10 cm by 10 cm by 10 cm) cubic cages placed close to (15 to 25 m) or far from (210 to 235 m) their respective towers. To avoid interference with EMF, we made cage frames with wood sticks and transparent tulle net. We set up 72 cages in each of the six study sites (three per EMF condition), 36 close to and 36 far from the transmission towers. We placed one honeybee per cage for 15 min under both experimental conditions, and then we removed the cages and stored them in an external place for a period of 60 min. We selected a period of 15 min of exposure to EMF since it is the maximal time recorded of honeybees flying over the flower patches (M.A.M-M.’s personal observation). We analyzed the level of Hsp70 at 0, 3, 5, 10, 30, and 60 min. The zero time consisted of putting the honeybee inside the cage and taking it out immediately, and it was considered as a control to evaluate handling stress. After exposure to each treatment, we stored all individuals in liquid nitrogen until we measured Hsp70 activity in the laboratory.

Second, to account for any potential effects of field environmental conditions on honeybee physiology and behavior, we exposed honeybees to an EMF in the laboratory using two custom-made solenoids as an EMF source. We made each solenoid with an iron pipe of 1.5 mm in thickness, 15 cm in diameter, and 20 cm in length. The pipe was covered by 300 turns of 1-mm-diameter varnished copper wire wound in two layers with no space between wires. To generate the EMF, each end of the copper wire was connected to the positive and negative terminals of a direct power supply capable of generating 24 V and 15 A (model CP20.241, Puls Dimension, Germany). To test the correct operation of the solenoids, we arranged four compasses around each solenoid and one inside the solenoid. The functioning of each solenoid was confirmed through the change induced on a compass, whose needle experienced a deviation from its magnetic north when the power source was switched on. The average intensity of the EMF inside the center of the solenoid was 7.8 ± 0.51 μT (*n* = 7 measures), a similar intensity to that recorded in the field at a distance of 25 to 30 m from the base of the transmission towers (i.e., 7.3 ± 0.78 μT). We placed two honeybees within each solenoid while one solenoid was turned on, and the other was off. After 10 s, we removed one honeybee from each solenoid and the second honeybee 3 min later; in both cases, we deposited the honeybees immediately in liquid nitrogen until protein extraction (Hsp70) or gene expression (indicated above). We selected those exposure times because they represent the minimum and maximum pollination times (contact between honeybee with California poppy) recorded for honeybees in the study site (M.A.M.-M.’s personal observation). We repeated this assay 25 times, alternating the solenoid assigned for the treatment on or off after each repetition to avoid any undesired effects generated by the devices.

To estimate the levels of stress exerted by EMF on honeybees and its effect on mRNA expression levels, we measured the expression of Hsp70 through enzyme-linked immunosorbent assay (ELISA) assays. For each honeybee, we homogenized head, thorax, and abdomen tissues in phosphate-buffered saline–azide–TAME buffer at 4°C (*34*). We centrifuged the homogenized tissue at 13,000*g* for 20 min at 4°C and used the supernatant as the protein source for ELISA assays. We used the DC (detergent-compatible) protein assay (Bio-Rad Inc., USA) to measure total soluble protein content supernatant at 750 nm in triplicate reactions using a microplate reader (Tecan, Model Sunrise, Switzerland). We estimated Hsp70 concentration using a monoclonal ELISA–modified method [see (*52*)]. We used the mouse antibovine HSP70 antibody (Sigma-Aldrich, St. Louis, Missouri, USA), a monoclonal antibody that recognizes a highly conserved region of HSP70 and heat shock cognate 70 protein. We read the absorbance of samples and Hsp70 standard at 450 nm with a microplate reader (Tecan, Model Sunrise, Switzerland). To assure statistically valid comparisons of experimental groups across multiple microplates, each microplate contained a bovine Hsp70 standard, and samples from different treatments were loaded on the microplates in a matched design to ensure equal replicates from each experimental group per microplate.

To estimate the effect on mRNA levels of candidate genes upon EMF exposure, honeybee heads were dissected on ice using a sterile scalpel. Total RNA was extracted using TRIzol (Invitrogen); integrity was assessed using a 1.1% agarose gel electrophoresis and quantificated by spectrophotometry at 260 nm (Epoch Microplate Spectrophotometer, BioTek) (*53*). DNA traces were removed by deoxyribonuclease (DNase) treatment using Turbo DNase (Ambion). Then, single-stranded cDNAs were synthesized using the SuperScript III Reverse Transcriptase System (Invitrogen). All procedures were conducted following the manufacturer’s instructions. Determination of relative transcript abundance was carried out by real-time quantitative polymerase chain reaction (qPCR) using specific primers. PCR reactions were carried out on a Mx3000 P qPCR system (Stratagene, California, USA) in triplicate (three technical replicates) under the following cycling conditions: 95°C for 10 min, 40 cycles of 95°C for 30 s, 56°C for 45 s, and 72°C for 40 s. Each PCR reaction contained 2 μl of diluted cDNA (2 ng; 1 ng/μl), 10 μl of Maxima SYBR Green PCR Master Mix (Thermo Fisher Scientific), 6.4 μl of nuclease-free water, and 0.8 μl of each specific primer (1.6 μl for both forward and reverse primers; 10 mM concentration). Expression data for each target gene were normalized using the ribosomal protein S5 of *A. mellifera* and calculated using the comparative 2 − ΔΔ*C*_T_ method (*54*).

### Effects of EMF on pollination services

California poppies were the most abundant flowering plant species in the study site across all plots. As this invasive species depends strongly on honeybees for seed production (*48*), we compared the abundance of honeybee individuals and their flower visitation rates between California poppy individuals growing close to and far from the electric transmission towers (turned on and turned off). We recorded flower visits over three consecutive sunny days during October 2015, i.e., the start of the austral spring. Each day, we set up eight 3 m–by–3 m plots (four plots for close and four plots for far) along four orthogonal directions (N, S, E, and W) surrounding the respective tower at each experimental site (three EMF-off and three EMF-on). Within each plot, we selected two California poppy individuals distanced by at least 2 m, each with contrasting floral display, to assess the effect of floral display size (low or high) on pollinator abundance and visitation frequency, both inside and outside of EMF influence (see below). Mean values of the low and high floral displays (number of flowers) across plots were 5.1 ± 1.6 individuals and 9.6 ± 1.3 individuals, respectively.

Although companion species included mainly shrubs and grasses, to avoid interferences with our study, we removed all flowering individuals from other species within a radius of 1.5 m around each target individual. We divided each observation day into 15 20-min observation periods conducted between 09:20 a.m. and 19:00 p.m. Therefore, we carried out a total of 1080 observation periods per each EMF condition (on-off). During each observation period, we observed all plots simultaneously with an experienced observer team. Each observer wore the same color of clothing to avoid interfering with pollinator visits. To estimate pollinator abundance, we recorded any insect present in the plot that could presumably visit flowers during the 20-min observation periods. We lastly considered them as flower visitors only if they touched anthers or stigma within flowers.

### Effects of EMF on plant reproduction

On the three experimental sites under active transmission towers (EMF-on), we evaluated the effect of the distance to the towers (close versus far), pollination type (naturally versus manually), and floral display (low versus high) on the reproduction of the California poppy. Within five new plots of 10 m by 10 m close to (15 to 25 m) and far from (210 to 235 m) the transmission towers, we randomly selected 20 individuals at each plot. We selected 10 individuals of low floral display and, separately, 10 individuals of high display. In this way, we sampled a total of 300 individuals of California poppy (20 individuals × 5 plots × 3 sites) at each distance from the towers. The mean number of flowers per individual for the small and large floral display size categories was 4.9 ± 1.1 and 9.6 ± 0.9, respectively.

From each individual plant, we randomly assigned two flowers to natural pollination and another two to assisted (hand) pollination. For the assisted pollination treatment, we pollinated once the focal flower with allogamous pollen carefully deposited on the stigma with a small brush. We collected the pollen from individuals located at least 15-m apart from focal plants to avoid fitness reductions due to inbreeding depression. We bagged hand-pollinated flowers immediately after withering to retain seeds. The natural pollination treatment consisted of naturally pollinated flowers bagged after withering to retain seeds using tulle net bags. After 2 months, we recovered all the bags with seeds dropped from the fruits and transported them to the laboratory to count the seeds. We averaged seed production of the two flowers assigned to the same treatment within a single individual and then calculated mean seed production for the different pollination treatments (natural or assisted), display levels (low or high), and spatial position relative to the towers (close or far).

### Effects of EMF on plant community

To assess whether the EMF intensity had any direct or indirect effect on vegetation, we recorded the presence and abundance of all plant species growing within 20 1 m–by–1 m quadrats located every 10 m along the same 200-m transects described for the EMF characterization. The community species richness, the overall plant abundances, and the relative abundance of *E. californica* within the local plant community were thus estimated around towers at both inactive (EMF-off) and active (EMF-on) transmission towers.

### Statistical analyses

To visualize the EMF around transmission towers at either inactive or active lines, we interpolated the respective field EMF intensities from the eight orthogonal transects sampled at each site using the *interp* function from the *akima* R package (*55*). To evaluate the effect of the EMF on honeybee physiology, we compared the temporal variation in Hsp70 close to and far from the transmission towers that were either inactive (EMF-off) or active (EMF-on) by fitting an LMM that incorporates the individuals within sites as a nested random factor. This was achieved using the *lme* function from the *nlme* R package (*56*). The significance of the random factor was assessed by comparing the full model (fixed + random factors) with a model without the inclusion of “individual in site” as a random factor. This was made with the *anova.lme* function also from the *nlme* R package (*56*). The effect of the EMF experimental exposure in the solenoid on the relative gene expression profiles of exposed and unexposed honeybees examined independently for each of the 14 selected genes by means of a paired *t* test. In addition, an evaluation of the level of expression of each gene relative to the internal normalizer (i.e., the “one-fold” reference) was also performed independently for each gene, under each exposure condition, through a one-sample *t* test under the null hypothesis of a true group mean equal to 1. Complementarily, we performed a two-way analysis of variance (ANOVA), also using R, to assess the effect of the EMF from the solenoid on the concentration of Hsp70 in laboratory honeybees. In this case, we included the solenoid condition (on-off) and the experimental time (start-end) as factors.

In addition, we used GAMMs to assess the effect of the distance (close-far) at which plants were from the EMF-on and EMF-off towers on the daily pattern of honeybee activity (i.e., honeybee local abundance and floral visits) around California poppy individuals with contrasting floral displays (low versus high). We took advantage of the ability of GAMMs to handle nonlinear relationships between variables and random factors in the data structure (i.e., plot and site). Analyses were performed with the *gamm* and *gam.check* functions from the *mgcv* R package (*54*). Complementarily, we conducted an additional LMM to assess, at the three sites of active electric transmission (EMF-on), the influence of the distance to the towers (close versus far) on the number of seeds produced by California poppy individuals and its interaction with their floral display (low versus high) and pollination type (natural or assisted). As above, plot and site were included as random factors during model fitting through the *lme* function from the *nlme* R package (*56*).

The effects of EMF on plant community (species richness and overall plant abundance) and on the abundance of the California poppy were assessed by fitting independent LMMs that include the activity of the line (EMF-off or EMF-on), the distance to the towers (close or far) and their interaction as fixed factors, and the cardinality of the transect (N, W, S, or E) and the site as random factors. For each response variable, the significance of the random factor was assessed by comparing the variance explained by the respective fitted model with that from a model without the inclusion of “cardinality in site” as a random factor. This was achieved through the *anova.lme* function, also from the *nlme* R package (*56*).

When required, we checked whether the model residuals fitted the normal distribution through Shapiro-Wilk tests. For the case of the significant LMM interactions, specific pairwise differences between treatments were assessed by comparing the least-square mean value for each group as allowed by the *emmeans* package (*57*). For the two-way ANOVA, a posteriori post hoc Tukey test was performed. By default, the first level of a categorical variable (alphabetically listed) is used in both LMM and GAMM analyses as the reference level for their internal comparisons; accordingly, EMF-off, “Distance-close,” and “Display-high” were the reference groups for the variables of “tower activity,” “distance to the towers,” and “floral display” in the referred analyses. Data visualization was achieved using functions from the plotly (*58*) and ggplot2 R packages (*59*).
