# Supplementary material for: Electromagnetic fields disrupt the pollination service by honeybees
Source: Sci Adv. 2023 May 12;9(19):eadh1455. doi: 10.1126/sciadv.adh1455 (PMC10181175; doi:10.1126/sciadv.adh1455)
Supplement: Supplementary file 1 — Figs. S1 and S2 Tables S1 to S10 [file sciadv.adh1455_sm.pdf]

Science Advances
AAAS

Supplementary Materials for

Electromagnetic fields disrupt the pollination service by honeybees

Marco A. Molina-Montenegro *et al.*

Corresponding author: Marco A. Molina-Montenegro, marco.molina@utalca.cl

Sci. Adv. **9**, eadh1455 (2023)
DOI: 10.1126/sciadv.adh1455

This PDF file includes:

Figs. S1 and S2
Tables S1 to S10

Supplementary Material

Supplementary Figure 1: Variation of Hsp70 concentration in the body tissue of individual honeybees experimentally exposed to an EMF for least for 3 minutes inside a solenoid that generates ~7.8 ± 0.51 μT. Individuals inside of a solenoid that were unexposed (EMF-off, grey) or exposed (EMF-on, blue) are shown. Error bars represent ±SD, significant differences between the group averages (Supplemental Table 5) were estimated by an *a-posteriori* Tukey Honest Significant Differences (HSD) test at the *p* level of 0.05, and are showed by different letters.

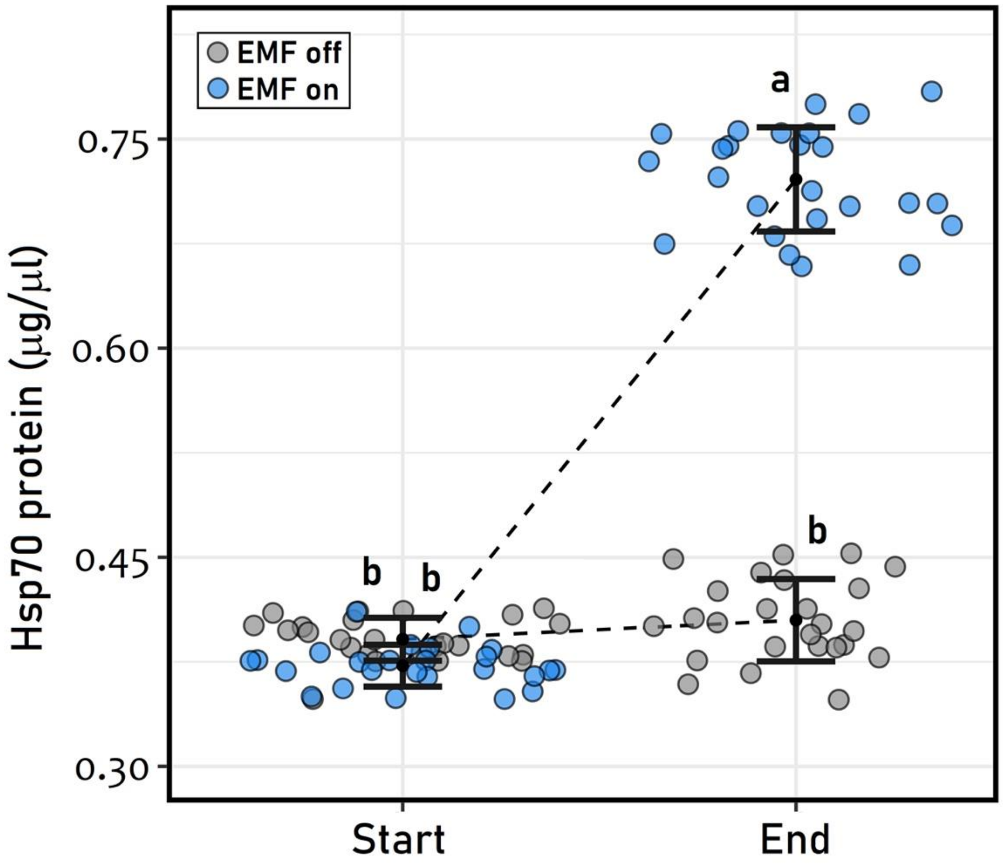

Supplementary Figure 2. Individual sampling counts(dots) of the abundance of *Apis melifera* individuals (a) and their 10-minute flower visit rates on California Poppy patches during daytime (b), as a function of the patch floral display (low: yellow/cyan; high: orange/blue) and the distance (orange/yellow: close = 15-25 m; blue/cyan: far = 210-235 m) at which floral patches were from the electrical current transmitting-towers. Observations were recorded during three consecutive days at towers that were not transmitting any current (EMF turned off) and at towers that did (EMF turned on). Colored dashed lines represent the smooth function of the General Additive Mixed Models (GAMMs) applied to the data. Shaded areas represent the respective 95% confidence interval for each fitted model. In general, overlapping intervals suggest non-significant differences between the involved groups.

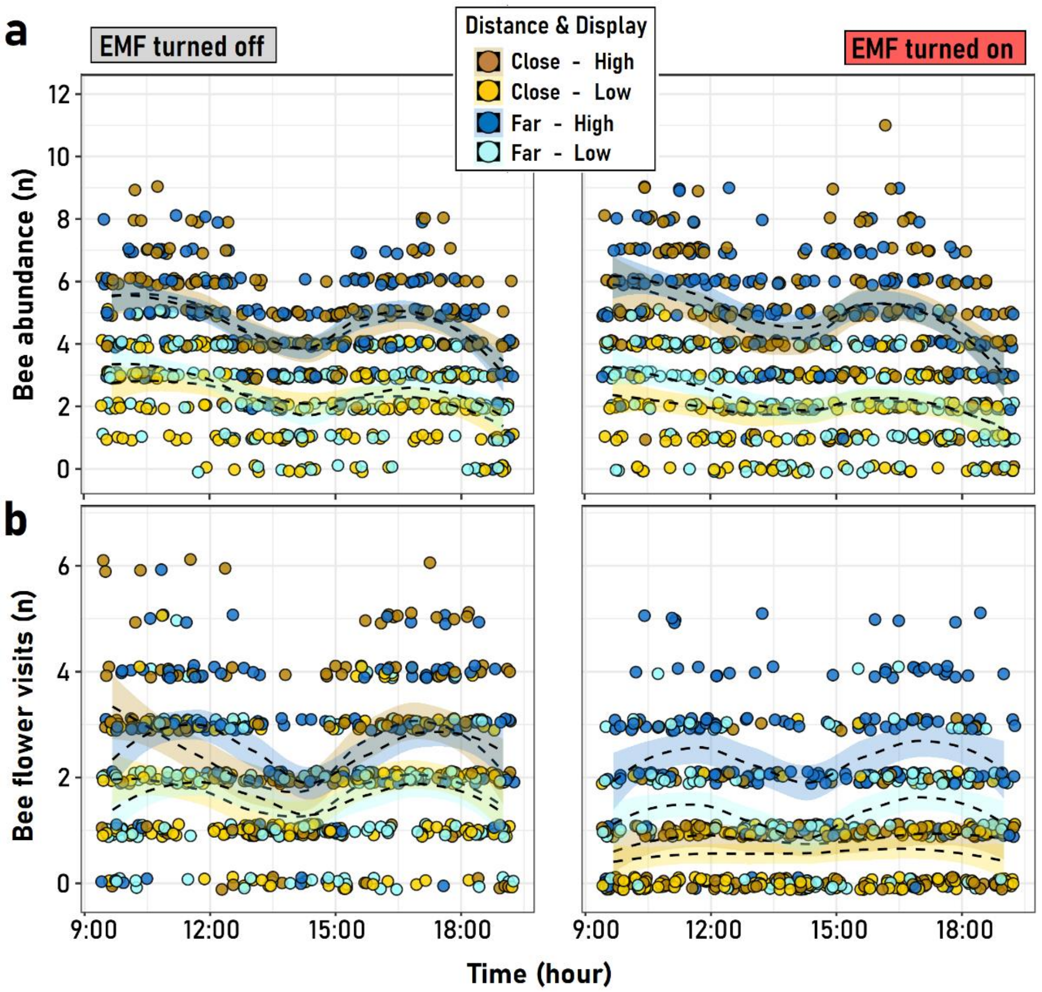

Supplementary Table 1. Linear mixed model (LMM) estimates describing the relation between the distance around the high voltage towers and the intensity of the electro-magnetic field (EMF) in transmission towers that were either active (EMF on) or inactive (EMF off, reference level). Both, fixed and random factors are shown. For the distance, the significance of a linear predictor suggests a relation between distance and EMF, while in a quadratic term it indicates the non-constancy of the amount of change along the sampled gradient. However, since both distance terms were only significant when interacting with “EMF activity”, the distance-EMF relation (in this case, an inverse one) must differ between “EMF activity” conditions. A significant negative coefficient for the “Distance1 x EMF” interaction points to a stepper (more negative) relation between variables among the “EMF-on” group. Regarding the random part of the model, the effect of the transect orientation (cardinality) nested in the sampled sites was not significant for explaining the data variance. Model coefficients (β), confidence intervals (C.I.), probability value (*p*), degrees of freedom (*d.f.*), likelihood ratio (L-ratio). Significance was considered at *p* values < 0.05 (in bold).

	**Predictor**	**β**	**C.I. (95%)**	***p***
**Electro-Magnetic Field (EMF)**	Intercept	10.21	[10.00 – 10.43]	0.151
	Distance1 (linear)	-0.13	[-0.14 – -0.12]	0.513
	Distance2 (quad.)	0.00042	[0.00039 – 0.00044]	0.694
	EMF activity (on)	11.72	[9.44 – 14.01]	**< 0.001**
	Distance1 x EMF activity (on)	-0.17	[-0.23 – -0.11]	**< 0.001**
	Distance2 x EMF activity (on)	0.0006	[0.0003 – 0.0009]	**< 0.001**
	**random effect**	***d.f.***	**L-ratio**	***p***
	Cardinality-in-site	9, 7	0.0312	0.9845

Supplementary Table 2. Effect of the distance to the transmission tower infrastructure (close: 15-25 m; far: 210-235 m) and the activity of the transmission line (EMF-off / EMF-on) in the temporal variation of the heat shock protein Hsp70 concentration among honeybees, as describe by a linear mixed model (LMM) fitted to field measurements. Both, fixed and random factors are shown. Among the fixed factors, the significance of the interaction terms "Distance x EMF" and "EMF x Time" suggests that the concentration of Hsp70 on the tissue of honeybees differently changes along time and distance, respectively, but only when the EMF is activated ("on"). This is also highlighted in the significance of the triple factor interaction. The random effect of the model (individuals nested within sites) was not significant. Model coefficient estimate (β), confidence intervals (C.I.), probability value (*p*), degrees of freedom (*d.f.*), likelihood ratio (L-ratio). Significance was considered at *p* values < 0.05 (in bold).

	**Predictor**	**Estimate (β)**	**95% C.I.**	***p***
**Honeybee HSP70 (at the field)**	Intercept	0.359	[0.345 – 0.374]	**< 0.001**
	Distance (far)	0.0009	[-0.019 – 0.021]	0.9231
	EMF (on)	0.163	[0.143 – 0.183]	**< 0.001**
	Time	-0.0004	[-0.0009 – 0.0001]	0.1136
	Distance x EMF	-0.158	[-0.187 – -0.130]	**< 0.001**
	Distance x Time	0.00001	[-0.0007 – 0.0007]	0.9771
	EMF x Time	0.0010	[0.0002 – 0.0017]	**0.0067**
	Distance x Tower x Time	-0.0011	[-0.0021 – -0.0001]	**0.0334**
	**random effect**	***d.f.***	**L-ratio**	***p***
	Individual-in-site	11, 9	1.563×10^{-8}	0.9999

Supplementary Table 3. Independent one-sample t-test performed on the relative expression values of 14 genes under each experimental treatment (EMF on - EMF off) to determine if they appeared overexpressed (> 1) or repressed (< 1) relative to the experimental normalizer gene *RS5* (Ribosomal protein). Significant differences from 1 (one-fold expression) were considered when $p > 0.05$ (in bold).

**EMF**	**Gene**	**Expression (mean)**	**t-statistic**	***d.f.***	**Low CI (95%)**	**High CI (95%)**	***p* val**
**Off**	***CaMKII***	1.383	20.245	9	1.340	1.426	**< 0.0001**
	CAT	1.062	5.014	9	1.034	1.090	**0.0007**
	CRY2	1.295	18.354	9	1.259	1.331	**< 0.0001**
	EGR1	1.112	8.127	9	1.081	1.143	**< 0.0001**
	For1	1.095	7.550	9	1.067	1.123	**< 0.0001**
	FTH1	1.490	17.446	9	1.426	1.554	**< 0.0001**
	GLOD4L	1.305	11.141	9	1.243	1.367	**< 0.0001**
	GstD1	1.096	6.635	9	1.063	1.129	**0.0001**
	HR38	1.206	13.477	9	1.171	1.240	**< 0.0001**
	HSP40	1.082	6.082	9	1.052	1.112	**0.0002**
	HSP70	1.072	5.441	9	1.042	1.102	**0.0004**
	SOD	1.144	7.562	9	1.101	1.187	**< 0.0001**
	TXNRD1	1.097	6.895	9	1.065	1.129	**0.0001**
	Vg	1.129	6.728	9	1.086	1.172	**0.0001**
**On**	***CaMKII***	0.530	-33.051	9	0.498	0.562	**< 0.0001**
	CAT	1.393	24.686	9	1.357	1.429	**< 0.0001**
	CRY2	0.593	-15.531	9	0.534	0.652	**< 0.0001**
	EGR1	0.454	-42.520	9	0.425	0.483	**< 0.0001**
	For1	0.579	-32.953	9	0.550	0.608	**< 0.0001**
	FTH1	1.542	21.933	9	1.486	1.598	**< 0.0001**
	GLOD4L	1.336	11.843	9	1.272	1.400	**< 0.0001**
	GstD1	1.460	18.920	9	1.405	1.515	**< 0.0001**
	HR38	0.707	-16.208	9	0.666	0.748	**< 0.0001**
	HSP40	2.037	44.713	9	1.985	2.089	**< 0.0001**
	HSP70	2.210	56.118	9	2.161	2.258	**< 0.0001**
	SOD	1.907	30.651	9	1.840	1.974	**< 0.0001**
	TXNRD1	1.348	18.947	9	1.306	1.390	**< 0.0001**
	Vg	0.445	-22.606	9	0.389	0.501	**< 0.0001**

Supplementary Table 4. Independent two-sample t-test performed between the relative expression values of 14 genes under either experimental treatment (EMF on - EMF off) to determine statistic differences in their expression levels. Significant differences between group averages were considered when $p > 0.05$ (in bold).

**Gene**	**Mean EMF Off**	**Mean EMF On**	**Mean diff.**	**t-statistic**	***d.f.***	**Low CI (95%)**	**High CI (95%)**	***p* val**
CaMKII	1.383	0.53	0.853	36.042	16.709	0.803	0.903	**< 0.0001**
CAT	1.062	1.393	-0.331	-16.421	16.961	-0.374	-0.288	**< 0.0001**
CRY2	1.295	0.593	0.702	22.835	14.931	0.636	0.768	**< 0.0001**
EGR1	1.112	0.454	0.658	34.909	17.906	0.619	0.698	**< 0.0001**
For1	1.095	0.579	0.516	28.775	17.996	0.478	0.554	**< 0.0001**
FTH1	1.49	1.542	-0.052	-1.39	17.713	-0.131	0.027	0.1818
GLOD4L	1.305	1.336	-0.031	-0.786	17.977	-0.114	0.052	0.4419
GstD1	1.096	1.46	-0.364	-12.866	14.664	-0.424	-0.304	**< 0.0001**
HR38	1.206	0.707	0.499	21.079	17.511	0.449	0.549	**< 0.0001**
HSP40	1.082	2.037	-0.955	-35.599	14.46	-1.012	-0.898	**< 0.0001**
HSP70	1.072	2.21	-1.138	-44.978	14.94	-1.192	-1.084	**< 0.0001**
SOD	1.144	1.907	-0.763	-21.682	15.363	-0.838	-0.688	**< 0.0001**
TXNRD1	1.097	1.348	-0.251	-10.849	16.856	-0.3	-0.202	**< 0.0001**
Vg	1.129	0.445	0.684	21.957	17.002	0.618	0.75	**< 0.0001**

Supplementary Table 5. Two-way ANOVA table examining the effect of the experimental 3-min EMF exposure (solenoid off vs. solenoid on) in the body concentration of heat shock protein Hsp70 among the two groups of honeybees. Degrees of freedom (*d.f.*), sum of squares (SS), mean squares (MS), Fisher statistic (*F*), p value (*p*). In both models, significant effects ($p < 0.05$) are highlighted in bold.

	**Factor**	***d.f.***	**SS**	**MS**	***F***	***p***
**Experimental Hsp70**	EMF	1	0.5522	0.5522	808.7	**< 0.001**
	Time	1	0.8212	0.8212	1203	**< 0.001**
	EMF x Time	1	0.7022	0.7022	1028.7	**< 0.001**
	residuals	96	0.0655	0.0007		

Supplementary Table 6: General Additive Mixed Model (GAMM) analysis on the diurnal (9:40am – 19:00 pm) distribution of honeybee recorded abundances over California Poppy plant patches surrounding electrical infrastructure (towers) that were either inactive (EMF-off), or actively transmitting electrical power (EMF-on). For either EMF condition, data was fitted as a function of the patch floral display (low or high) and the distance (close = 15-25 m; far = 210-235 m) at which floral patches were from the transmission towers. The fitted GAMM estimates for the parametric coefficients are shown together with the significance of the smooth term for each experimental group, which if not-significant denoted a null improvement of the model fitting with the referred smoothed term (*i.e.* the underlying trend is better explained with a linear predictor only). The level of reference used for each factor is also shown between parentheses. Significance was considered at probability (p) values < 0.05 (in bold), estimated degrees of freedom (edf), t-statistic (t), F-statistic (F).

	**Factor**	**Estimate**	**std. error**	***t***	***p***
**Honeybee Abundances (EMF-off)**	Intercept	1.541	0.029	52.17	**< 0.0001**
	Distance (far)	-0.009	0.040	-0.29	0.819
	Flower display (low)	-0.796	0.040	-19.64	**< 0.0001**
	Distance x flower display	-0.076	0.057	1.32	0.186
	**Approximate significance of smooth terms**		**edf**	***F***	***p***
	Close/ High flower display		7.09	17.25	**< 0.0001**
	Close/ Low flower display		4.08	6.19	**< 0.0001**
	Far/ High flower display		6.59	17.39	**< 0.0001**
	Far/ Low flower display		5.94	11.40	**< 0.0001**

	**Factor**	**Estimate**	**std. error**	***t***	***p***
**Honeybee Abundances (EMF-on)**	Intercept	1.804	0.036	50.17	**< 0.0001**
	Distance (far)	-0.053	0.046	-1.16	0.246
	Flower display (low)	-0.968	0.046	-20.92	**< 0.0001**
	Distance x flower display	-0.051	0.065	-0.79	0.429
	**Approximate significance of smooth terms**		**edf**	***F***	***p***
	Close/ High flower display		6.91	14.13	**< 0.0001**
	Close/ Low flower display		4.28	2.64	0.105
	Far/ High flower display		6.24	15.63	**< 0.0001**
	Far/ Low flower display		4.25	8.04	**0.0005**

Supplementary Table 7. Estimates of the general additive mixed model (GAMM) analyses on the diurnal (9:40am – 19:00 pm) distribution of honeybee (*A. melifera*) individual visit rates (10 min) on flowers of California Poppy at individual patches surrounding electrical infrastructure (towers) that were either inactive (EMF-off), or actively transmitting electrical current (EMF-on). For either EMF condition, data was fitted as a function of the patch floral display (low or high) and the distance (close = 15-25 m; far = 210-235 m) at which floral patches were from the transmission towers. The fitted GAMM estimates for the parametric coefficients are shown together with the significance of the smooth term for each experimental group, which if not-significant denoted a null improvement of the model fitting with the referred smoothed term (*i.e.* the underlying trend is better explained with a linear predictor only). The level of reference used for each factor is also shown between parentheses. Significance was considered at probability (*p*) values < 0.05 (in bold), estimated degrees of freedom (edf), t-statistic (*t*), F-statistic (*F*).

	**Factor**	**Estimate**	**std. error**	***t***	***p***
**Honeybee Flower visit rates (EMF-off)**	Intercept	0.853	0.031	26.91	**< 0.0001**
	Distance (far)	0.026	0.038	0.66	0.505
	Flower display (low)	-0.266	0.039	-6.85	**< 0.0001**
	Distance x flower display	-0.050	0.055	-0.91	0.364
	**Approximate significance of smooth terms**		**edf**	***F***	***P***
	Close/ High flower display		5.83	9.65	**< 0.0001**
	Close/ Low flower display		5.24	4.17	**0.0008**
	Far/ High flower display		6.22	8.34	**< 0.0001**
	Far/ Low flower display		5.20	3.95	**0.0012**

	**Factor**	**Estimate**	**std. error**	***t***	***P***
**Honeybee Flower visit rates (EMF-on)**	Intercept	0.340	0.023	14.50	**< 0.0001**
	Distance (far)	0.512	0.030	16.87	**< 0.0001**
	Flower display (low)	-0.095	0.030	-3.12	**0.0017**
	Distance x flower display	-0.249	0.043	-5.81	**< 0.0001**
	**Approximate significance of smooth terms**		**edf**	***F***	***P***
	Close/ High flower display		1.00	0.55	0.455
	Close/ Low flower display		1.00	0.11	0.734
	Far/ High flower display		6.57	8.52	**< 0.0001**
	Far/ Low flower display		5.99	7.14	**< 0.0001**

Supplementary Table 8. Effect of the distance to active (EMF-on) high-voltage electric transmission towers on the number of seeds produced by *E. californica* individuals, its interaction with the level of flower display (high / low) and pollination type (natural or assisted). Both, fixed and random factors are showed. The significance of the interaction term Distance x Pollination suggests that the number of seeds depends on the pollination type only at certain distances. In regard of the random part of the model, the effect of the nested sampling (plot in sites) was not relevant for the explained variance. Model coefficients (β), confidence intervals (C.I.), probability value (*p*), degrees of freedom (*d.f.*), likelihood ratio (L-ratio). Significance was considered at *p* values < 0.05 (in bold).

	**Predictor**	**β**	**C.I. (95%)**	***p***
***E. californica* seed production**	Intercept	49.13	[47.95 – 50.32]	**< 0.001**
	Distance	-23.86	[-25.38 – -22.36]	**< 0.001**
	Flower display	-3.66	[-5.18 – -2.16]	**< 0.001**
	Pollination type	-5.73	[-7.24 – -4.22]	**< 0.001**
	EMF x Flower display	0.13	[-2.01 – 2.27]	0.903
	EMF x Pollination	25.53	[23.40 – 27.67]	**< 0.001**
	Flower display x Pollination	1.73	[-0.40 – 3.87]	0.114
	EMF x Flower display x Pollination	0.60	[-2.42 – 3.62]	0.698
	**random effect**	***d.f***	**L-ratio**	***p***
	Plot in site	11, 9	2.0085	0.3663

Supplementary Table 9. Effect of the distance to the electric transmission infrastructure (high-voltage towers) and their EMF activity ("off" or "on") on the richness of the local plant community, its overall abundance, and the relative abundance of *E. californica*. Both, fixed and random factors are showed in each case. Despite the significance of the random components of the model, contrary to the distance, the influence of the EMF activity was significant among all variables, always with negative coefficients, suggesting an overall reduction in all variables when the EMF activity is "on", relative to the "off" condition. For the plant richness, the significant positive linear coefficient (β) implies a slight trend to increase plant richness with EMF intensity, however the significant negative quadratic term ("Distance 2") suggests that a bell-shaped non-linear relation may fit better to the overall data. In the case of both, plant abundance and the relative abundance of *E. californica*, the distance as a main factor resulted non-significant, however their interactions did. This suggests that the effect of the distance on the measured variables was significantly different between EMF conditions (on – off). The positive coefficients of the significant (linear) interaction "Distance1 x EMF activity" in both models suggests that abundances presented a significantly higher rate of change (increase) with increasing distances from the towers in the "on" condition (relative to "off", the baseline). The significant interaction with the quadratic model suggests that different bell-shaped non-linear relations (quadratic parameters) might correspond to each EMF group. Model coefficients (β), confidence intervals (C.I.), probability value (*p*), degrees of freedom (*d.f.*), likelihood ratio (L-ratio). Significance was considered at *p* values < 0.05 (in bold).

	**Predictor**	**β**	**C.I. (95%)**	***p***
**Plant species richness**	Intercept	4.212	[3.85 – 4.57]	**< 0.001**
	Distance (linear)	0.016	[0.009 – 0.023]	**< 0.001**
	Distance 2 (quad.)	-0.00007	[-0.0001 – -0.00003]	**< 0.001**
	EMF activity (on)	-1.14	[-1.55 – -0.73]	**< 0.001**
	Distance x EMF activity (on)	0.028	[0.018 – 0.039]	**< 0.001**
	Distance 2 x EMF activity (on)	-0.00015	[-0.0002 – -0.00014]	**< 0.001**
	**random effect**	***d.f.***	**L-ratio**	***p***
	Cardinality-in-site	9, 7	11.28	**0.0036**
**Overall plant abundance**	**Predictor**	**β**	**C.I. (95%)**	***p***
	Intercept	15.38	[14.69 – 16.08]	**< 0.001**
	Distance (linear)	-0.003	[-0.018 – 0.001]	0.605
	Distance 2 (quad.)	0.00003	[-0.0003 – 0.0001]	0.326
	EMF activity (on)	-10.92	[-11.75 – -10.09]	**< 0.001**
	Distance x EMF activity (on)	0.18	[0.16 – 0.20]	**< 0.001**
	Distance 2 x EMF activity (on)	-0.0006	[-0.0007 – -0.00005]	**< 0.001**
	**random effect**	***d.f.***	**L-ratio**	***p***
	Cardinality-in-site	9, 7	9.88	**0.0071**
***E. californica* relative abundance**	**Predictor**	**β**	**C.I. (95%)**	***p***
	Intercept	62.6282	[59.06 – 66.19]	**< 0.001**
	Distance (linear)	-0.01266	[-0.060 – 0.035]	0.6042
	Distance 2 (quad.)	0.00006	[-0.0001 – 0.0003]	0.6148
	EMF activity (on)	-50.07544	[-52.71 – -47.37]	**< 0.001**
	Distance x EMF activity (on)	0.63913	[0.57 – 0.70]	**< 0.001**
	Distance 2 x EMF activity (on)	-0.00155	[-0.0018 – -0.0012]	**< 0.001**
	**random effect**	***d.f.***	**L-ratio**	***p***
	Cardinality-in-site	9, 7	47.68	**0.001**

Supplementary Table 10: Chi-square test of independence for the abundances and effective floral visit rates of each insect taxa between patches of *E. californica* close or far electric line infrastructures that were either inactive (off) or actively (on) transmitting high-voltage current. χ^2: chi-square statistic, d.f.: degrees of freedom, *p*: probability value. Significant (non-independent) relations are highlighted in bold ($p < 0.05$). In addition, abundance and visits to flowers of California poppy is presented as the number of individuals as well as the percentage of different taxa recorded in the study site. A lepidopteran unidentified taxon observed occasionally at the field was not present among the recorded floral visitors.

Variable	EMF	Family	Species	χ^2	*p*
**Abundance**	**off**	Hymenoptera	*Apis melifera*	0.031	0.858
		Hymenoptera	*Bombus dahlbomii*	0.047	0.828
		Coleoptera	*Astylus* sp.	0.161	0.688
		Diptera	*Dilophus* sp.	0.642	0.427
		Lepidoptera	Un-identified	0.000	1.000
	**on**	Hymenoptera	*Apis melifera*	2.767	0.096
		Hymenoptera	*Bombus dahlbomii*	0.0072	0.787
		Coleoptera	*Astylus* sp.	0.164	0.691
		Diptera	*Dilophus* sp.	0.529	0.466
		Lepidoptera	Un-identified	3.001	0.083
**Visits**	**off**	Hymenoptera	*Apis melifera*	0.001	0.999
		Hymenoptera	*Bombus dahlbomii*	0.085	0.769
		Coleoptera	*Astylus* sp.	0.126	0.721
		Diptera	*Dilophus* sp.	0.000	1.000
		Lepidoptera	Un-identified	-	-
	**on**	Hymenoptera	*Apis melifera*	187.99	**< 0.001**
		Hymenoptera	*Bombus dahlbomii*	14.785	**< 0.001**
		Coleoptera	*Astylus* sp.	9.523	**0.002**
		Diptera	*Dilophus* sp.	7.538	**0.006**
		Lepidoptera	Un-identified	-	-

Variable	EMF	Family	Species	*Number of individuals*	*Percentage*
**Abundance (turned-off)**	**close**	Hymenoptera	*Apis melifera*	1274	20.5
		Hymenoptera	*Bombus dahlbomii*	168	2.7
		Coleoptera	*Astylus* sp.	75	1.2
		Diptera	*Dilophus* sp.	25	0.4
		Lepidoptera	Un-identified	4	0
	**far**	Hymenoptera	*Apis melifera*	1265	20.3
		Hymenoptera	*Bombus dahlbomii*	172	2.8
		Coleoptera	*Astylus* sp.	80	1.3
		Diptera	*Dilophus* sp.	31	0.5
		Lepidoptera	Un-identified	4	0

**Abundance (turned-on)**	**close**	Hymenoptera	*Apis melifera*	1233	20.0
		Hymenoptera	*Bombus dahlbomii*	174	2.8
		Coleoptera	*Astylus* sp.	80	1.3
		Diptera	*Dilophus* sp.	31	0.5
		Lepidoptera	Un-identified	0	0
	**far**	Hymenoptera	*Apis melifera*	1317	21.2
		Hymenoptera	*Bombus dahlbomii*	169	2.7
		Coleoptera	*Astylus* sp.	75	1.2
		Diptera	*Dilophus* sp.	37	0.6
		Lepidoptera	Un-identified	3	0

**Visits (turned-off)**	**close**	Hymenoptera	*Apis melifera*	778	27.6
		Hymenoptera	*Bombus dahlbomii*	54	1.9
		Coleoptera	*Astylus* sp.	34	1.2
		Diptera	*Dilophus* sp.	20	0.6
		Lepidoptera	Un-identified	0	0
	**far**	Hymenoptera	*Apis melifera*	778	27.6
		Hymenoptera	*Bombus dahlbomii*	51	1.8
		Coleoptera	*Astylus* sp.	37	1.3
		Diptera	*Dilophus* sp.	20	0.6
		Lepidoptera	Un-identified	0	0
**Visits (turned-on)**	**close**	Hymenoptera	*Apis melifera*	254	9.0
		Hymenoptera	*Bombus dahlbomii*	17	0.6
		Coleoptera	*Astylus* sp.	11	0.4
		Diptera	*Dilophus* sp.	6	0.2
		Lepidoptera	Un-identified	0	0
	**far**	Hymenoptera	*Apis melifera*	671	23.8
		Hymenoptera	*Bombus dahlbomii*	48	1.7
		Coleoptera	*Astylus* sp.	31	1.1
		Diptera	*Dilophus* sp.	20	0.6
		Lepidoptera	Un-identified	0	0
